# Supplementary material for: Prebiotic Treatment During Pregnancy Prevents Social Deficits Associated with Autism Spectrum Disorder-like Behavior Induced by Maternal Immune Activation
Source: Microorganisms. 2025 Dec 26;14(1):60. doi: 10.3390/microorganisms14010060 (PMC12843959; doi:10.3390/microorganisms14010060)
Supplement: Supplementary file 1 [file microorganisms-14-00060-s001.zip › microorganisms-3714547-supplementary.pdf]

microorganisms

MDPI

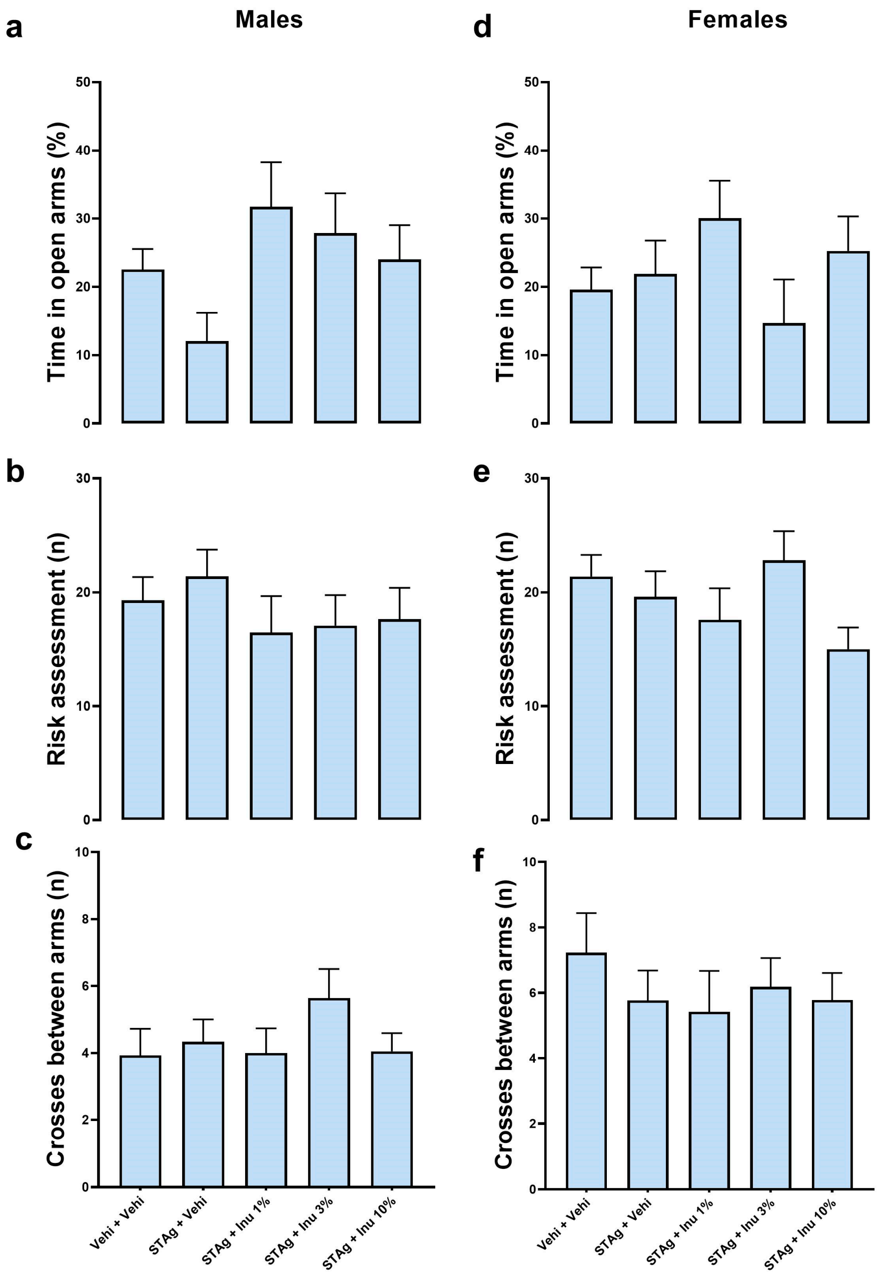

Figure S1. Elevated plus maze test. (a-c) males. (d-f) females. a/d = time spent in open arms (%). b/e = risk assessment behaviors (n). c/f = crossings between closed arms (n). Bars represent M±SEM. INU = Inulin; STAg = Soluble Toxoplasma gondii Antigen; PBS = Phosphate-buffered saline. N = 9-20.

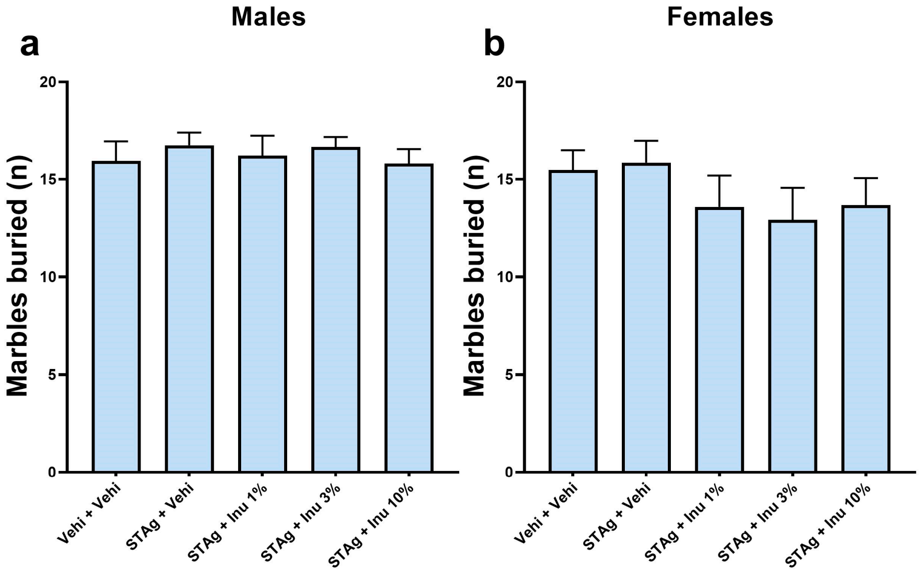

Figure S2. Marble buring test. a = Number of marbles buried by males (n). b = Number of marbles buried by females (n). Bars represent M±SEM. INU = Inulin; STAg = Soluble Toxoplasma gondii Antigen; PBS = Phosphate-buffered saline. N = 9-20.

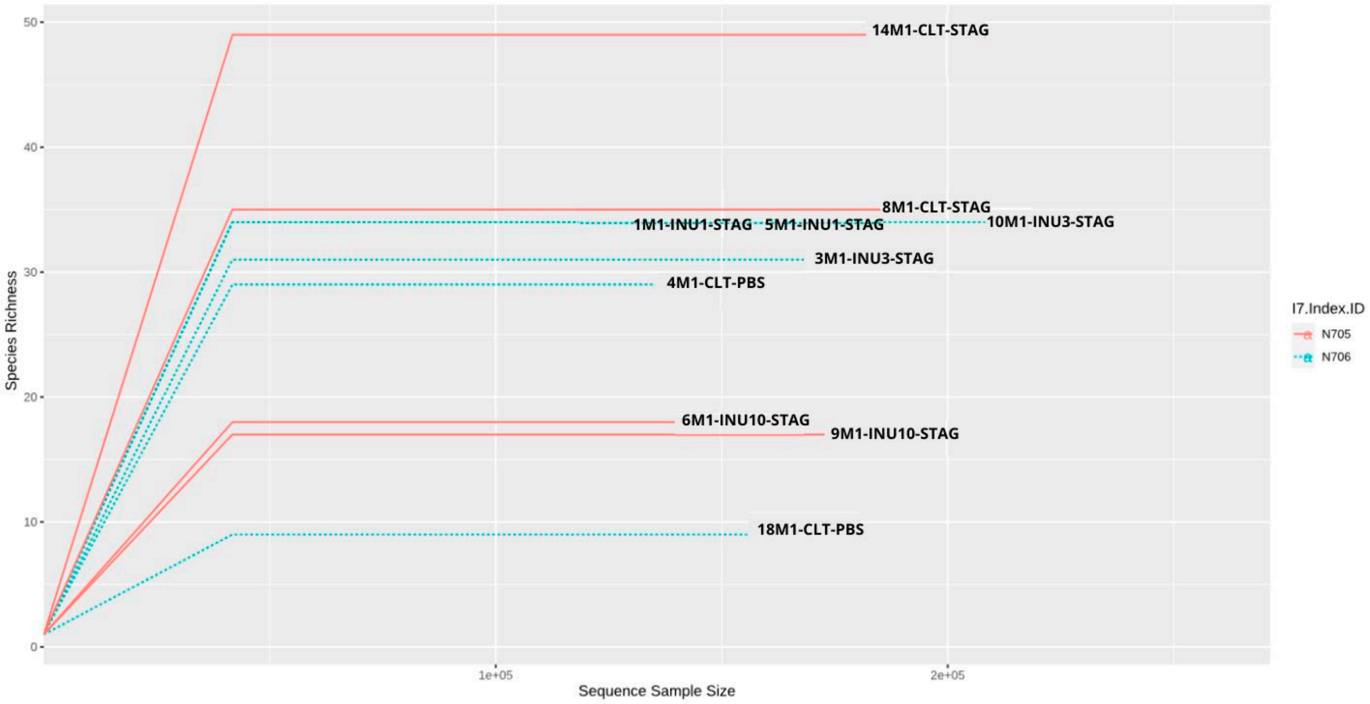

Figure S3. Rarefaction curve. The x-axis represents the number of sequences sampled, and the y-axis indicates the species richness detected, as estimated by the Chao1 index.
